# The effect of an electronic cognitive aid on the management of ST-elevation myocardial infarction during caesarean section: a prospective randomised simulation study

**DOI:** 10.1186/s12871-017-0340-4

**Published:** 2017-03-20

**Authors:** Michael St.Pierre, Bjoern Luetcke, Dieter Strembski, Christopher Schmitt, Georg Breuer

**Affiliations:** Anästhesiologische Klinik, Universitätsklinikum Erlangen, Krankenhaustrasse 12, 91054 Erlangen, Germany

**Keywords:** Checklist, Myocardial infarction, Obstetrics, Patient safety, Simulation

## Abstract

**Background:**

Cognitive aids have come to be viewed as promising tools in the management of perioperative critical events. The majority of published simulation studies have focussed on perioperative crises that are characterised by time pressure, rare occurrence, or complex management steps (e.g., cardiac arrest emergencies, management of the difficult airway). At present, there is limited information on the usefulness of cognitive aids in critical situations with moderate time pressure and complexity. Intraoperative myocardial infarction may be an emergency to which these limitations apply.

**Methods:**

Anaesthetic teams were allocated to control (no cognitive aid; *n* = 10) or intervention (cognitive aid provided; *n* = 10) groups. The primary aim of this study was to compare cognitive aid versus memory for intraoperative ST-elevation myocardial infarction (STEMI) management in a simulation of caesarean delivery under spinal anaesthesia. We identified nine evidence-based metrics of essential care from current guidelines and subdivided them into mandatory (high level of evidence; no interference with surgery) and optional (lower class of recommendation; possible impact on surgery) tasks. Six clinically relevant tasks were added by consensus. Implementation of these steps was measured by scoring task items in a binary fashion (yes/no). The interval between the diagnosis of STEMI and the first contact with the cardiac catheterisation lab was measured. To determine whether or not the cognitive aid had prompted an action, participants from the cognitive aid group were interviewed during debriefing on every single treatment step. At the end of the simulation, session participants were asked to complete a survey.

**Results:**

The presence of the cognitive aid did not shorten the time interval until the cardiac catheterisation lab was contacted. The availability of the cognitive aid improved task performance in the tasks identified from the guidelines (93% vs. 69%; *p* < 0.001) as well as overall task performance (87.5% vs. 59%; *p* < 0.001). The observed difference in performance can be attributed to the use of the cognitive aid, as performance from memory alone would have been comparable across both groups. Trainees appeared to derive greater benefit from the cognitive aid than did consultants and nurses.

**Conclusions:**

The management of intraoperative ST-elevation myocardial infarction can be improved if teams use a cognitive aid. Trainees appeared to derive greater benefit from the cognitive aid than did consultants and nurses.

**Electronic supplementary material:**

The online version of this article (doi:10.1186/s12871-017-0340-4) contains supplementary material, which is available to authorized users.

## Background

Crisis-related cognitive aids (CA) commonly referred to as a “crisis checklist” [1], “emergency manual” [2], or “emergency quick reference guide” [3, 4], provide prompts for and reviews of critical steps during time-sensitive high-stress situations. Their goal is to offset the large cognitive load involved in crisis management and to help translate best practices for patient care during acute events [5]. Ideally, CAs anticipate common pitfalls of the particular emergency, provide prioritised and explicit instructions to prevent them, and contain important local information (e.g. phone numbers, depositories of critical drugs, pre-calculated drug dosages, etc.). While CAs have come to be viewed as one of the most promising tools in the management of perioperative critical events [6], others have recommended a cautious approach as as the complexity of pathophysiological changes, the unpredictability of therapeutic interventions, and interference with the surgical procedure may not be adequately covered by emergency manuals [7].

Intraoperative myocardial infarction, regularly covered by crisis checklists and emergency manuals [2, 3, 8, 9], may be an emergency to which these limitations apply. Critical treatment steps (e.g., 12-lead ECG within 10 min, reperfusion therapy within 60 min [10]) may be difficult to perform in a patient still undergoing surgery. In addition, some recommended critical tasks (e.g., aspirin i.v., heparin i.v., intravenous nitrates) may interfere with the surgical procedure and have to be negotiated with the surgeon instead of simply being correctly performed.

Several case reports have reported on ST-elevation myocardial infarction (STEMI) during elective caesarean section under spinal anaesthesia [11, 12]. The unlikeliness of such an event in an otherwise healthy woman may delay timely treatment in the form of percutaneous coronary intervention (PCI) when managed from memory alone. We hypothesised that the use of a CA on intraoperative STEMI would a) accelerate transfer to the cardiac catheterisation lab and b) improve complete consideration of all recommended tasks [10] while at the same time permit clinicians to negotiate potentially harmful measures with their surgical colleague.

The primary aim of this study was to compare the use of a CA versus memory for intraoperative STEMI management in a simulation of caesarean delivery under spinal anaesthesia. Although participants had to diagnose the presence of STEMI, the primary aim was the treatment of myocardial infarction and not the timing or pathway to diagnosis. The secondary aim was to determine the perception of participants on the usefulness and clinical relevance of the CA.

## Methods

### Participants

**Fig. 1:**
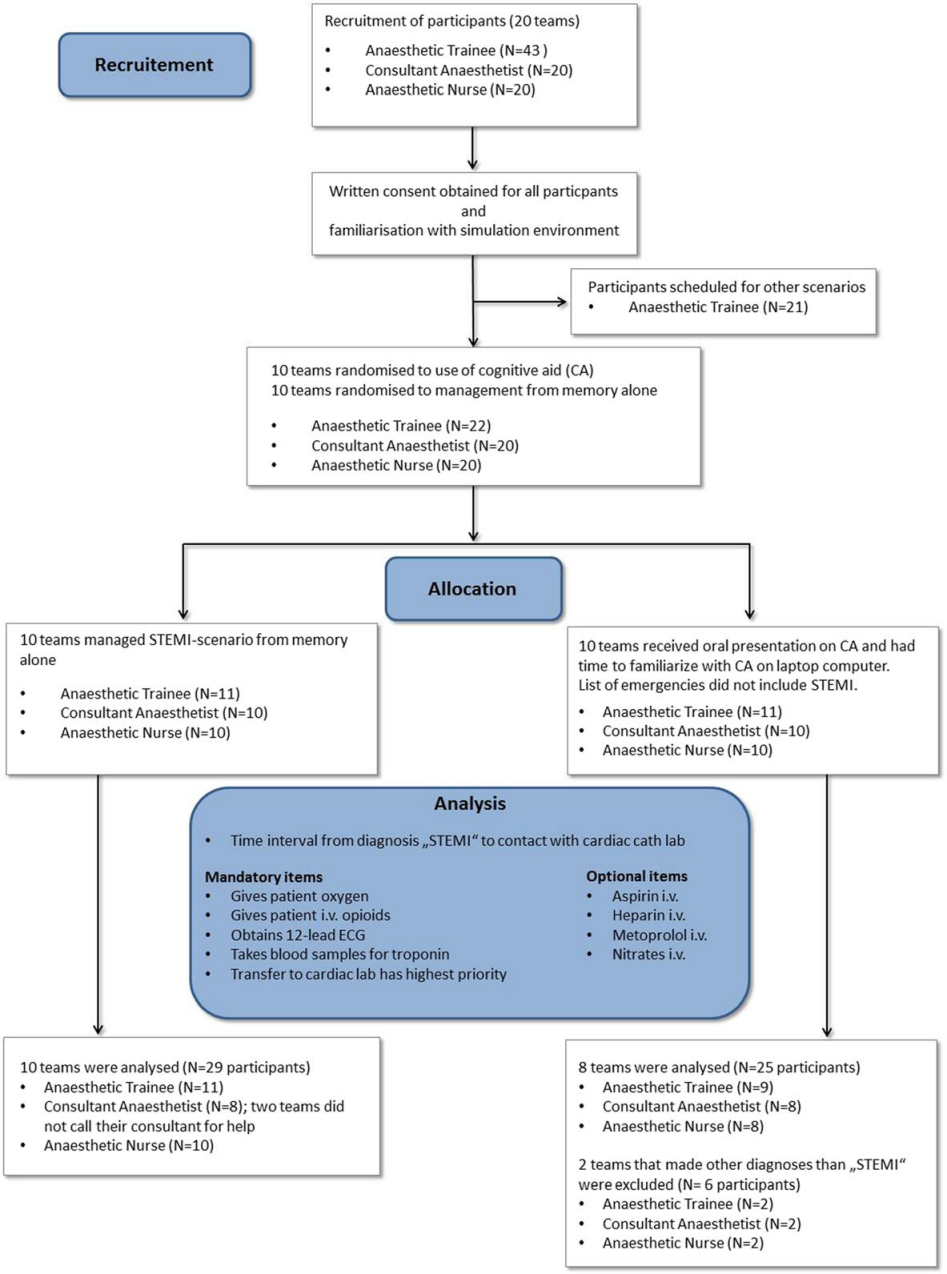
CONSORT *flow chart* of recruitment, randomisation, and analysis. CA; cognitive aid

After obtaining approval of the study protocol by the ethics committee of the Friedrich-Alexander University Erlangen–Nuremberg (reference number 270_15), we enrolled 83 participants into this prospective, randomised single-blinded controlled trial (Fig. 1). Participants were part of a 20 days institutional training program at the authors’ department. Scheduling for each day reflected the actual role composition of anaesthetic teams commonly found in German anaesthesia departments. Teams consisted of 1–2 anaesthetic trainees, a consultant anaesthetist and an anaesthetic nurse.

Written informed consent was obtained from all participants prior to the second scenario. Participants from both groups were briefed on the scenario with a standard amount of information. The script for the intervention group was supplemented with a final passage reminding participants to use the CA once a diagnosis had been made.

### Study protocol

The first two scenarios of the day (i.e., STEMI during C-section, intraoperative complication during hysteroscopy) were randomly alternated using a web-based tool (www.randomizer.com). All participants managed their first scenario from memory alone (control group). Following standardised educational intervention, participants were able to access the CA during the second scenario (cognitive aid (CA) group). Standardised educational intervention consisted of a didactic and practical training. The aim of the didactic training was to familiarise participants with the concept of CAs and with the alternative approaches of using them during an emergency (i.e.; in a prospective “challenge-response” manner or in a retrospective “do-verify” manner). During practical training, participants were given opportunity to familiarise with the structure and content of the CA by trying it out on a laptop computer. In this training version the list of emergencies did not include the emergency to follow in the next scenario.

Performance was scored from the point when the participant(s) articulated a diagnosis of either acute coronary syndrome (ACS) or STEMI. The study protocol required that every scenario where participants made diagnoses other than ACS or STEMI had to be excluded from further analysis.

### Cognitive aid

**Fig. 2:**
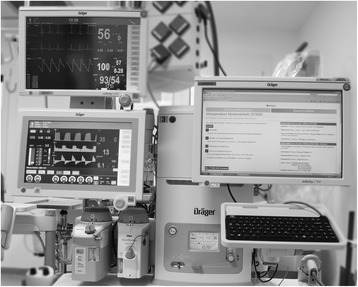
Location of anaesthesia information management system with web-based cognitive aid. Placement provides easy accessibility and consistent location, without interfering with routine *work flow*. The layout was adapted from a paper based template available from Ariadne Labs [1]

The CA was available as a set of HTML5-pages throughout the simulation and accessible via web-browser on the institution’s anaesthesia information management system mounted on the anaesthesia machine (Fig. 2). Each HTML5-page started with a header containing the identification and description of the emergency, followed by a bold type statement regarding the management priority. Capitalising on the monitors landscape format we chose a two-columned layout with action steps on the left and reference information on the right side as others have done [1, 8, 13]. The design of the CA followed recommendations for CAs in medicine [14] as well as design guidance for electronic emergency checklists in aviation [15, 16]. A prototype was developed and tested by the authors before its first use during the study trial. The medical content of the CA on “Intraoperative Myocardial Infarction” was developed by reviewing guidelines from recognised bodies [10, 17, 18] as well as commercial [8] and open access emergency manuals [1–3, 9]. The final action items for the checklist were selected by consensus among the authors and adapted to local conditions. An Additional file 1 shows the translated text of the original German version of the CA on “Intraoperative Myocardial Infarction” (see Additional file 1). The CA used during the simulation contained a list with 36 adult and paediatric emergencies to ensure that participants selected the diagnosis from clinical cues and not out of convenience due to only having one scenario available.

### Scenario

The crisis scenario was based on published case reports of acute myocardial infarction during caesarean section under spinal anaesthesia [11, 12]: Shortly after delivery of a healthy infant with surgery still underway an otherwise healthy patient developed chest pain and shortness of breath. The symptoms the patient complained about were typical for STEMI (e.g., severe pain radiating to the neck, shoulder and left arm) to optimise the likelihood that participants would make the correct diagnosis. Prior to the onset of symptoms the patient had received 3 IE oxytocin as slow intravenous bolus followed by a maintenance infusion of 10 IE oxytocin as mandated by the institutional protocol. Symptoms increased over the next 5–10 min with a concomitant drop in saturation (target SpO_2_: 93%), tachycardia (target heart rate: 130 bpm) and a hypertensive blood pressure response (target blood pressure: 150/90 mmHg). Target values were chosen to allow the full range of treatment interventions for STEMI [10]. ST-segment elevation increased at the same rate and was clearly visible on the monitor. Haemodynamic variables and cardiac rhythms were programmed into a manikin-based simulator (SimMan; Laerdal Norway). The protocol was pretested before study commencement using nonparticipating subjects with the aim of improving clinical cues for the participants. The results served to refine the programming and simulation script.

In the scenario, anaesthetic care was handed off to the primary provider by a confederate anaesthetist after spinal anaesthesia had been established. No husband or other family member was in the room with the patient. The consultant anaesthetist was sequestered in a separate room and could be summoned for help by the primary anaesthetist if requested. During the scenario three confederates played the roles of obstetricians and circulating nurses. They did not assist the participants in implementing the critical steps. However, when the anaesthetist raised the issue of nitroglycerine and its impact on uterine contraction or of intravenous aspirin/heparin, the confederate obstetricians agreed to a low dose infusion of nitroglycerine or to a low dose of aspirin/heparin.

To facilitate an intraoperative diagnosis, the study design allowed that a 12-lead ECG could be obtained and interpreted without delay as soon as participants requested it. A feature of the SimMan-monitor made it possible to display a 12-lead ECG with significant ST-segment elevations (>0.25 mV) in five contiguous leads (V2–V6).

### Assessment tools and scoring

Team performance was assessed using a scenario-specific 15-item checklist. The items were generated by identifying nine evidence-based metrics of essential care for acute myocardial infarction from the ESC-guidelines [10] and by subdividing them by consensus into five mandatory and four optional tasks (Table 1). Mandatory tasks were those with a high level of evidence and class of recommendation (IA-IC) that did not interfere with the surgical procedure. Optional tasks were defined as measures that either had a lower class of recommendation (IIa) or that could possibly impact the surgical procedure (e.g., i.v. heparin, i.v. nitrates) and therefore should be negotiated with the obstetrician. Six additional clinically relevant tasks were added by consensus by the authors (e.g., “calls for help early”). We did not develop a scoring system by assigning points for the various treatment steps, as reported by other research teams [19]. Instead, we confined ourselves to scoring task items in a binary fashion (yes/no) and to weighting them equally. While correct performance was the criterion for mandatory tasks, an optional task item was considered completed if the anaesthetist discussed the measure with team members or the obstetrician. We recorded how often the monitor with the CA was accessed during the scenario either by an individual or by the entire team. With the exception of the interval between the diagnosis of STEMI and the first contact with the cardiac catheterization lab, we did not measure any time frame within which key processes had to be performed, as others have done [13].

**Table 1:** Specific task performance and task consideration data

	Class and Level^b^	Cognitive Aid (*n* = 8)	No Cognitive Aid (*n* = 10)	Differences in Adherence
Mandatory tasks (5)				
• Gives patient oxygen	IC	8 (100.0)	7 (70.0)	30%
• Gives patient i.v. opioids (fentanyl or morphine)	IC	8 (100.0)	9 (90.0)	10%
• Obtains 12-lead ECG as soon as possible	IB	8 (100.0)	8 (80.0)	20%
• Takes or orders blood sampling for troponin	IC	5 (62.5)	8 (80.0)	17.5%^a^
• Transfer to cardiac lab has highest treatment priority	IA	7 (87.5)	8 (80.0)	7.5%
Optional tasks (4)				
• Considers or gives aspirin i.v.	IB	8 (100.0)	8 (80.0)	20%
• Considers or gives unfractionated heparin i.v.	IC	8 (100.0)	9 (90.0)	10%
• Considers or gives i.v. metoprolol	IIa B	7 (87.5)	1 (10.0)	77.5%
• Considers or gives i.v. nitrates	IIa C	8 (100.0)	4 (40.0)	60%
Clinically relevant tasks by consensus (6)				
• Calls for help early	n/a	8 (100.0)	8 (80.0)	20%
• Informs surgeon	n/a	8 (100.0)	10 (100.0)	0%^a^
• Participant considers: Does surgeon consent to treatment?	n/a	8 (100)	9 (90.0)	10%
• Participant considers: Epidural-/spinal anaesthesia as contraindication?	n/a	6 (75.0)	0 (0.0)	75%
• Checks Hb and considers transfusion (aim Hb 7–9 g/dl)	n/a; ESA-recommendation [3]	1 (12.5)	0 (0.0)	12.5%
• Participant knows phone number of cath lab	n/a	8 (100.0)	0 (0.0)	100%

Values in column 3 (Cognitive Aid) and 4 (No Cognitive Aid) are number of teams correctly performing or considering the task (%). Values in column 5 are differences in adherence to the individual task between the cognitive aid group and the control group

*Abbreviations*: *n/a* not applicable

^a^With the exception of the tasks “Takes or orders blood sampling for troponin” and “Informs surgeon” the adherence for tasks was higher in the cognitive aid group

^b^Class of recommendation and level of evidence as stated in the ESC Guidelines for the management of acute myocardial infarction in patients presenting with ST-segment elevation [10]

In the intervention group, we attributed an action or a consideration to the use of a CA if a) the anaesthetist read out aloud the treatment step and then either gave the order or started to discuss the measure with team members or the obstetrician or b) an individual’s action immediately followed reading the cognitive aid or c) the trainee or consultant stated during debriefing that the action or consideration had been in response to an item of the cognitive aid. As key processes were binary outcomes (yes/no) we assumed that adjudications of actions could be easily made.

A list of six survey items regarding the usefulness and clinical relevance of CAs was generated from a literature review by one of the authors (MS). Face validity and content validity were assessed via discussion by the five authors. Responses to survey questions were binary (agree/don’t agree) (Table 2). Because all anaesthetists and anaesthetic nurses were candidates of the institutional training program, we were unable to pilot test the survey on a subpopulation within our department prior to the study.

**Table 2:** Survey questions regarding usefulness and clinical relevance of cognitive aid (Translated from German)

	Trainee (*n* = 9)	Consultant (*n* = 8)	Nurse (*n* = 8)
1. I found the CA helpful because it reminded me of treatment steps I otherwise might have forgotten.	6 (66)	2 (25)	3 (37.5)
2. I found the CA helpful because we could check our treatment steps for completeness.	8 (89)	6 (75)	5 (62.5)
3. I found the CA helpful because it promoted team discussion of our treatment steps.	5 (55)	4 (50)	4 (50)
4. I would appreciate the introduction of the CA into daily practice.	7 (87.5)	6 (75)	3 (37.5)
5. I would not use the CA in an intraoperative emergency, but I could imagine that inexperienced colleagues may benefit from using it.	0 (0)	0 (0)	0 (0)
6. For a successful implementation of the CA it would be necessary to establish a ‘code reader’ who would guide the team through all treatment steps.	2 (22)	3 (33)	2 (25)

*Abbreviations*: *CA* cognitive aid

Items were scored in a binary fashion (agree/don’t agree)

Values are number of participants (%)

### Data collection

Multiscreen synchronised video recordings were taken of all 20 scenarios and were available for offline evaluation. To determine whether or not the CA had prompted an action, we interviewed participants from the intervention group during debriefing on every single treatment step. We asked them whether they would have performed the task in any case or whether reading the CA had reminded them of the measure. At the end of the simulation session participants were asked to complete the survey. We explained to the participants that the term ‘code reader’ (question 6) denotes a dedicated person who assists the team leader by reading critical steps aloud from the CA and then acknowledging completion of each step [20].

### Evaluators

Task performance data of all scenarios was evaluated by one of the five authors. Video data of all 20 scenarios was reviewed and mandatory and optional tasks were scored by a single study team member (MS). Ten random scenarios were independently reviewed by a second team member (GB).

### Statistics

Data were analysed with the use of SPSS software version 21.0 (IBM). All reported *p* values are two-sided, and *p*-values of less than 0.05 were considered statistically significant. Participant characteristics and time intervals were compared with a two-tailed *t*-test. Task performance data (yes/no) were compared with Fisher’s exact tests, responses to survey questions (yes/no) were analysed by applying the chi-squared test if applicable. Interrater agreement on the scoring of mandatory and optional task completion in 10 random scenarios was assessed using Cohens’ kappa statistic.

### Cognitive errors analysis

If participants made diagnoses other than ACS or STEMI and then failed to adjust their initial diagnosis in the further course we interviewed them during debriefing about their decision-making process and related their responses to the most frequent cognitive errors [21].

## Results

Twenty teams (22 anaesthetic trainees, 20 consultants, and 20 anaesthetic nurses) participated in the study. Two teams from the intervention group made the diagnosis of “amniotic fluid embolism”. In both of these teams, no critical steps for STEMI were performed. As intended by the study design, both scenarios were excluded from further analysis. In both groups there was one scenario where a second trainee was called for help. Two consultants from the control group were not summoned for help by the primary anaesthetist. As a result, data from 18 teams (20 anaesthetic trainees, 16 consultants, and 18 anaesthetic nurses) were analysed (Fig. 1). There were no group differences in terms of years of clinical experience (Table 3).

**Table 3:** Participant characteristics of the randomly assigned groups: Years of clinical experience

Characteristics	Cognitive Aid Group (*n* = 25)	No Cognitive Aid Group (*n* = 29)
Consultant	10.9 (±6.2) yrs	12.6 (±5.4) yrs
Anaesthetist Trainee	4.5 (±3.2) yrs	4.1 (±2.3) yrs
Anaesthetic Nurse	11.8 (±8.6) yrs	8.7 (±8.2) yrs

Data are mean (SD)

The inter-rater reliability for the scoring of task completion and task consideration showed good agreement (κ = 0.76). Given that key processes were binary outcomes (yes/no) consensus between raters was easily achieved for any instance of initial disagreement.

The presence of the CA did not shorten the time interval from the first diagnosis of “STEMI” until the cardiac catheterisation lab was contacted (340 ± 128 s with CA vs. 295 ± 157 s without CA; p = 0.55). The availability of the CA improved task performance in the nine tasks identified from the ESC guidelines [67/72 (93%) vs. 62/90 (69%) tasks; *p* < 0.001] as well as overall task performance [105/120 (87.5%) vs. 89/150 (59%); *p* < 0.001)]. With the exception of the tasks “Takes or orders blood sampling for troponin” and “Informs surgeon” the adherence for individual tasks was higher in the CA group (7.5 to 77.5% difference; Table 1). Only one team in the CA group checked the haemoglobin concentration as a contributing factor for coronary ischemia in patients undergoing open surgery. The data collected during debriefing indicate that the observed difference in performance can be attributed to the use of the CA, as performance from memory alone would have been comparable across both groups (Table 4).

**Table 4:** Without the cognitive aid both groups would have performed equally

	Cognitive Aid (*n* = 8) Debriefing	No Cognitive Aid (*n* = 10) Scenario
	From memory alone	From memory alone
• Calls for help early	8 (100.0)	8 (80.0)
• Gives patient oxygen	7 (87.5)	7 (70.0)
• Gives patient i.v. opioids (fentanyl or morphine)	8 (100.0)	9 (90.0)
• Obtains 12-lead ECG as soon as possible	8 (100.0)	8 (80.0)
• Takes or orders blood sampling for troponin	5 (62.5)	8 (80.0)
• Transfer to cardiac lab has highest treatment priority	6 (75.0)	8 (80.0)
• Informs surgeon	8 (100)	10 (100.0)
• Considers or gives aspirin i.v.	8 (100.0)	8 (80.0)
• Considers or gives unfractionated heparin i.v.	8 (100.0)	9 (90.0)
• Considers or gives i.v. metoprolol	3 (37.5)	1 (10.0)
• Considers or gives i.v. nitrates	5 (62.5)	4 (40.0)
• Checks Hb and considers transfusion	0 (0.0)	0 (0.0)
• Participant considers: Does surgeon consent to treatment?	8 (100.0)	9 (90.0)
• Participant considers: Epidural-/spinal anaesthesia as contraindication?	0 (0.0)	0 (0.0)
• Participant knows phone number of cath lab	0 (0.0)	0 (0.0)

*Abbreviations*: *CA* cognitive aid

Data in column 2 (Cognitive Aid) were collected during debriefing, where participants declared whether a task had been performed or considered from memory alone or in response to an item of the cognitive aid. Frequencies in column 3 (No Cognitive Aid) describe task performance during the scenario

Values are number of teams (%)

In seven out of eight scenarios (87.5%) the physician initiated the use of the CA, whereas the anaesthetic nurse opened the website only once (12.5%). In five out of eight scenarios (62.5%) the CA was used in a challenge-response mode, while three out of eight teams (37.5%) used the CA to verify task completion after execution. We found no association between the frequency with which teams accessed the CA and their STEMI treatment performance.

Participant survey responses pertaining to the simulation experience with the CA and to the usefulness and clinical relevance of the CA are detailed in Table 2. Trainees found the CA more helpful with regard to the availability of critical information than consultants and anaesthetic nurses. Only a minority of participants voted for the implementation of a designated ‘code reader’.

## Discussion

Encouraged by promising results from industries such as nuclear power or civil aviation who have long embraced emergency procedure manuals or checklists to manage high-stakes, high-stress situations, anaesthesia has witnessed growing interest in CAs that can counteract the possible deleterious effects of stress on human cognitive functions. Simulation testing plays a vital role in both assessing the usefulness and clinical relevance of a particular CA, as well as in the iterative refinement of usability and accessibility [5]. The majority of published simulation studies have focussed on perioperative crises that are characterised by time pressure, rare occurrence, or complex management steps (e.g., anaphylaxis, cardiac arrest emergencies, the management of a difficult airway, malignant hyperthermia crises, and the management of local anaesthetic toxicity [22]). To our knowledge, the present study is the first to evaluate the usefulness and clinical relevance of a CA in the management of an intraoperative STEMI. Traditional management of these emergencies is often based on a limited set of established tasks, at times memorised with an acronym (e.g., ‘MONA’: “morphine, oxygen, nitroglycerine, aspirin”). Our results suggest that treatment from memory alone does not ensure complete adherence to recommended treatment steps: with regard to the treatment steps of ‘MONA’ the task adherence rate ranged from 40% (nitrates) to 90% (i.v. opioids) (Table 1). In contrast, the availability of a CA increased adherence rate to 100% in all four critical tasks.

**Fig. 3:**
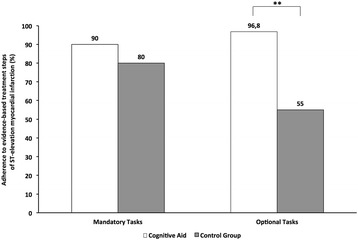
Adherence to critical treatment steps in ST-elevation myocardial infarction as defined by the guidelines of the European Society of Cardiology [10]. Data on frequency is taken from Table 1: Mandatory (5 evidence-based items) and optional (4 evidence-based items) management tasks. ** *p* < 0.002

We deliberately chose arterial hypertension and tachycardia as haemodynamic target values to give participants the opportunity to apply the full range of treatment options for STEMI, as recommended by the task force of the European Society of Cardiology (e.g., intravenous beta blockers, intravenous nitrates) [10]. While only half of the teams of the intervention group considered correcting the hemodynamic situation, treatment by the intervention group was characterised by almost complete guideline adherence (Fig. 3). Interviews during debriefing revealed that guideline adherence can be attributed to the availability of a CA: without the CA, the performance from memory alone by teams in the intervention group would have been comparable to the performance of the control group (Table 4). Besides guiding participants on the basis of current evidence-based practices, the CA also helped to anticipate common pitfalls, i.e. whereas 75% of teams in the intervention group discussed the pros and cons of giving heparin to a patient who had just received neuraxial blockade, no team in the control group considered this contraindication. Our findings contribute to our knowledge of CAs in so far as they underscore the fact that teams benefit from being reminded of additional treatment options, contraindications, and important local information (e.g., phone number of the cath lab). Results from a recent survey on emergency manual use during actual critical events indicate that these benefits are not confined to simulation testing but translate into the clinical environment [23]. In contrast to Harrison et al. [19] who found a relationship between the frequency of CA use and malignant hyperthermia treatment, we were unable to correlate the frequency with which teams accessed the CA and their STEMI treatment performance. We think that this difference can be explained by clinicians’ familiarity with the aetiology and treatment of STEMI as compared to malignant hyperthermia. Contrasting these results, it appears that CAs can demonstrate their potential particularly in rare emergencies that require a complex and specific treatment plan that has to be implemented quickly and efficiently.

Early provision of percutaneous coronary intervention (PCI) is the treatment priority for STEMI and a predictor of outcomes. Previous studies have recommended that the design of CAs should include a clear statement concerning management priorities of the respective emergency [19]. We designed the CA with a headline that emphasised transfer to the catheterisation lab as the highest priority (Appendix 1). We had hypothesised that the use of a CA with a clear definition of treatment priorities would shorten the time from the diagnosis of STEMI to the first phone call to the cardiac lab. To our surprise, this was not the case. Instead, the majority of participants from both groups prioritised immediate transfer to PCI and contacted the cardiologist within five minutes. We believe that these results do not call into question the design considerations concerning a clear statement about treatment priorities, but rather suggest that participants of both groups were well aware of the importance of PCI in the treatment of STEMI.

Despite efforts to provide as many salient clinical cues as possible to optimise the likelihood of participants making the correct diagnosis, two teams did not diagnose STEMI. Instead, they committed themselves to a diagnosis of amniotic fluid embolism. Errors that lead to an incorrect diagnosis are caused by faulty thought processes and are called ‘cognitive errors’. The past decade has witnessed a growing interest in understanding the cognitive underpinnings of decision making in anaesthesia, because cognitive errors can contribute significantly to medical mishaps [24, 25]. Cognitive errors are not knowledge deficits but origin from systematic deviations from rational decision-making. These systematic deviations (e.g. heuristics) are neither inherently good nor bad. Whether or not they simplify clinical decision-making or lead the clinician astray depends on the context of the application and the structure of the environment [21]. As some degree of heuristic decision making is inevitable in a dynamic critical situation, the cognitive error lies not in the nature of the preliminary diagnosis itself, but in the fact that intuitive judgments and biases are not submitted to verification by deliberate consideration. This override function of conscious deliberation, which can prevent humans from taking immediate action on first impressions, appears to be a critical feature in good decision-making [21]. Because cognitive debiasing strategies can be learned [26], thinking mistakes are thought to be potentially preventable.

In our study, the two teams that made diagnoses other than ACS or STEMI perceptually locked on two salient features of the initial clinical presentation (i.e. dyspnea, hypoxaemia) and then failed to adjust their initial prognosis in the light of later and contradicting information (i.e. ST-segment elevation, arterial hypertension) (‘anchoring’). Both teams closed their decision-making process as soon as the first plausible explanation for the patients’ deterioration had been found (‘premature closure’/’search satisficing’). Video analysis revealed that participants consistently sought pieces of information that reinforced the present hypotheses rather than refuting it (‘confirmation bias’). These findings are in accordance with observations from other simulation studies in anaesthesia, where ‘anchoring’, ‘premature closure’, and ‘confirmation bias’ were frequently observed cognitive errors [25].

When responding to the survey questions, anaesthetic trainees valued the possibility of using the CA to check treatment steps for completeness more than consultants or anaesthetic nurses (Table 2). We were pleasantly surprised that no participant in general, and in particular no consultant declared that they were beyond the need of a CA in the case of an intraoperative emergency. However, the positive appraisal does not justify the conclusion that the performance during simulation will actually translate into clinical practice. Results from surveys suggest that there is a gap between the intention to use a CA and the actual management of clinical critical events. While more than 80% of anaesthetists declare that they would use CAs if they were accessible at their institution as few as 7% of clinicians actually use an available CA [27, 28]. Institutions that take great care to thoughtfully implement CAs into their organisational complex work environment appear to achieve a more frequent use of CAs [23].

Although we chose a caesarean section as scenario for intraoperative STEMI, we believe that our results can be generalised beyond obstetric anaesthesia. Not only the management of perioperative crises characterised by great time pressure and rare occurrence, but also the management of more common emergencies with moderate time pressure (e.g. STEMI) can be substantially improved if teams use a CA.

There are a number of study limitations. Our findings should be interpreted as results of a simulation study and not of a field study with real parturients. Accepting the result of interventions tested by means of simulation invariably carries risks [29]. Given the fact that the study was conducted during the annual 20 days institutional training program we did not perform an a priori sample size calculation. Rather, we used a convenience sample, targeting all participating consultants, anaesthetic trainees, and anaesthetic nurses. As a result, the number of participants leaves the study underpowered to detect a difference for each individual task listed in Table 1. Instead, we compared task adherence on the level of mandatory and optional tasks as well on the level of overall task performance.

Neither participants nor our video reviewers could be blinded to the CA use. As a result, the video review and the scoring of mandatory and optional tasks are at risk of being biased. However, we believe that there was only limited room for personal interpretation because specific task performance and task consideration data had binary outcomes. The time frame within which STEMI was diagnosed is clinically unrealistic. Because our study design required an unproblematic intraoperative electrocardiographic diagnosis for further assessment of task performance, participants could obtain and interpret a 12-lead ECG as soon as they requested it. As a matter of course, the instantaneous availability of a 12-lead-ECG certainly promoted the rapid detection of significant ST-elevations in contingent leads. As a result, the diagnosis of STEMI and transfer to PCI most certainly will be delayed in real intraoperative myocardial infarctions. We chose the scenario of STEMI during elective caesarean section because we believed that the unlikeliness of such an event in an otherwise healthy woman would delay transfer to PCI when managed from memory alone and because the recommended critical tasks (e.g., anticoagulation, intravenous nitrates) would interfere with the surgical procedure and make negotiation with the surgeon mandatory.

The short time interval in the control group from the first diagnosis of “STEMI” until the cardiac catheterisation lab was contacted may not be representative of anaesthetists in general but may partially be explained by the characteristics of our anaesthesia department, as anaesthetic trainees and consultants have an additional qualification as German prehospital emergency physicians (“Notarzt”) and are routinely dispatched to preclinical emergencies. As a result, participants from the control group might have been more familiar with PCI as the treatment priority for STEMI than anaesthetists whose workplace is the operating theatre or a surgical intensive care unit. Although a team of experienced simulation instructors developed the post-simulation survey, it did not undergo validation testing. In addition, the small sample size precluded statistical analysis (chi-squared test) for the majority of questions. Nonetheless, we believe that the questions were context-relevant and were suited to elicit the perception of participants on the usefulness and clinical relevance of the CA.

## Conclusions

The management of intraoperative ST-elevation myocardial infarction can be improved if teams use a cognitive aid. Trainees appeared to derive greater benefit from the cognitive aid than did consultants and nurses.

## Additional file

Additional file 1: Text of cognitive aid (Translated from German, not original formatting). The layout was adapted from a template available from Ariadne Labs [1]. (DOCX 26 kb)

## Abbreviations

ACS: Acute coronary syndrome
AHF: Acute heart failure
CA: Cognitive aid
ECG: Electrocardiogram
ESC: European Society of Cardiology
HTML5: Hyper Text Markup Language version 5
ICU: Intensive care unit
PCI: Percutaneous coronary intervention
PEEP: Positive endexpiratory pressure
STEMI: ST-elevation myocardial infarction
