# Supplementary material for: The effect of an electronic cognitive aid on the management of ST-elevation myocardial infarction during caesarean section: a prospective randomised simulation study
Source: BMC Anesthesiol. 2017 Mar 20;17:46. doi: 10.1186/s12871-017-0340-4 (PMC5359845; doi:10.1186/s12871-017-0340-4)
Supplement: Additional file 1: — Text of cognitive aid (Translated from German, not original formatting). The layout was adapted from a template available from Ariadne Labs [1]. (DOCX 26 kb) [file 12871_2017_340_MOESM1_ESM.docx]

**Additional file 1:** Text of cognitive aid (Translated from German, not original formatting). The layout was adapted from a template available from Ariadne Labs [1]

**Intraoperative Myocardial Infarction (STEMI)**

| ECG: New ST-segment elevation, new left bundle block, chest pain (in awake patient) |
| --- |

**Highest priority: Immediate transfer to cardiac catheter lab**

**START..--.**

_❶_ **Call for help**

As soon as ST-segment elevation is detected: immediately call your consultant

_❷_**Verify suspected diagnosis**

Expand monitor view or obtain 12-lead ECG a soon as possible. Print rhythm strip.

_❸_**Inform surgeon**

_❹_**Ensure Oxygenation**

- Increase F_i_O_2_ to 100% (SpO_2_ > 94%, P_a_O_2_>80 mmHg)
- Check for anaemia and consider transfusion if Hb <8g/dl

**Haemodynamically STABLE..--.**

_❶_ **Reduce stress response**

- Adequate depth of anaesthesia?
- Adequate analgesia? In awake patients with regional anaesthesia consider morphine or other narcotic i.v.

_❷_**Reduce heart rate** (aim:60-80 bpm)

- Consider metoprolole i.v.

_❸_**Improve coronary perfusion**

- Consider nitroglycerin infusion

_❹_**Antithrombotic therapy and anticoagulation**

- Consider aspirin i.v.
- Consider heparin

**CAVE:**

- Surgeon consents to treatment?
- No epidural- /spinal anaesthesia?

_❺_**Stabilize cardiac rhythm**

- In case of new supraventricular or ventricular arrhythmias: Consider amiodarone

_❻_**Contact** **catheterization laboratory as soon as possible**

- Phone number: 45300 (available 24/7)

_❼_**Plan transport as soon as possible**

- Transport via corridor system: Patient bed at holding area?
- Transport with ambulance: Call 112

**Haemodynamically UNSTABLE..--.**

_❶_ **Stabilize haemodynamics**

- In case of hypovolemia: (positive Trendelenburg test): repeat. volume bolus (250 ml saline)
- Consider dobutamine infusion
- Consider norepinephrine infusion
- Consider epinephrine in case of refractory hypotension.

_❷_ **Stabilize cardiac rhythm**

- In case of bradycardia: Go to bradycardia
- In case of new supraventricular or ventricular arrhythmias
  - Consider amiodarone
  - Consider synchronized cardioversion

_❸_ In case of **ventricular tachycardia/ventricular fibrillation**

- Go to ventricular tachycardia/ventricular fibrillation

_❹_**Antithrombotic therapy and anticoagulation**

- Consider aspirin i.v.
- Consider heparin

**CAVE:**

- Surgeon consents to treatment?
- No epidural- /spinal anaesthesia?

_❺_**Advanced haemodynamic monitoring**

- Consider TEE
- Consider arterial line
- Consider central venous access

_❻_**Contact** **catheterisation laboratory as soon as possible**

- Phone number: 45300 (available 24/7)

_❼_**Plan transport as soon as possible**

- Transport via corridor system: Patient bed at holding area?
- Transport with ambulance: Call 112
